# Rapidly Progressive Osteonecrosis of the Humeral Head Following Arthroscopic Superior Capsule Reconstruction: A Case Report

**DOI:** 10.1002/ccr3.73340

**Published:** 2026-08-18

**Authors:** Suguru Mikami, Yohei Shimada, Koh Terauchi, Shu Somemura, Yosuke Kano, Naoki Haraguchi

**Affiliations:** ^1^ Department of Orthopedic Surgery St. Marianna University School of Medicine Kawasaki Japan

**Keywords:** arthroscopy, case report, rapidly destructive osteonecrosis, shoulder, superior capsule reconstruction

## Abstract

Rapidly destructive osteonecrosis of the humeral head can develop after superior capsule reconstruction despite intact graft healing. New or worsening shoulder pain with rapid functional decline several months postoperatively should raise suspicion for osteonecrosis and prompt early imaging and intervention.

## 1 Introduction

Rapidly destructive osteonecrosis (RDON) of the humeral head after arthroscopic shoulder surgery is extremely rare. Dilisio et al. [1] reported an incidence of 0.28% after arthroscopic rotator cuff repair (ARCR), suggesting vascular compromise of the humeral head as a potential mechanism.

Superior capsule reconstruction (SCR) has been increasingly utilized for irreparable massive rotator cuff tears to restore joint stability and shoulder elevation. However, reports of RDON occurring after SCR are extremely limited, and its pathophysiology has not been sufficiently discussed.

We present a rare case of rapidly destructive humeral head osteonecrosis after SCR requiring conversion to reverse total shoulder arthroplasty (RTSA), and discuss possible mechanisms by referring to previous reports of RDON following ARCR.

## 2 Case Presentation/Examination

A 68‐year‐old male carpenter sustained a fall and presented with right shoulder pain and elevation difficulty. He was initially treated at a previous hospital, where conservative management failed to improve symptoms, leading to referral for surgical treatment.

His medical history included diabetes mellitus and coracoid transfer surgery performed 40 years earlier for recurrent shoulder dislocation. He denied smoking or alcohol use.

On initial examination, active forward elevation of the right shoulder was 45° (contralateral side 150°), abduction 45° (150°), external rotation 20° (45°), and internal rotation to the L3 level (contralateral L1).

**FIGURE 1:**
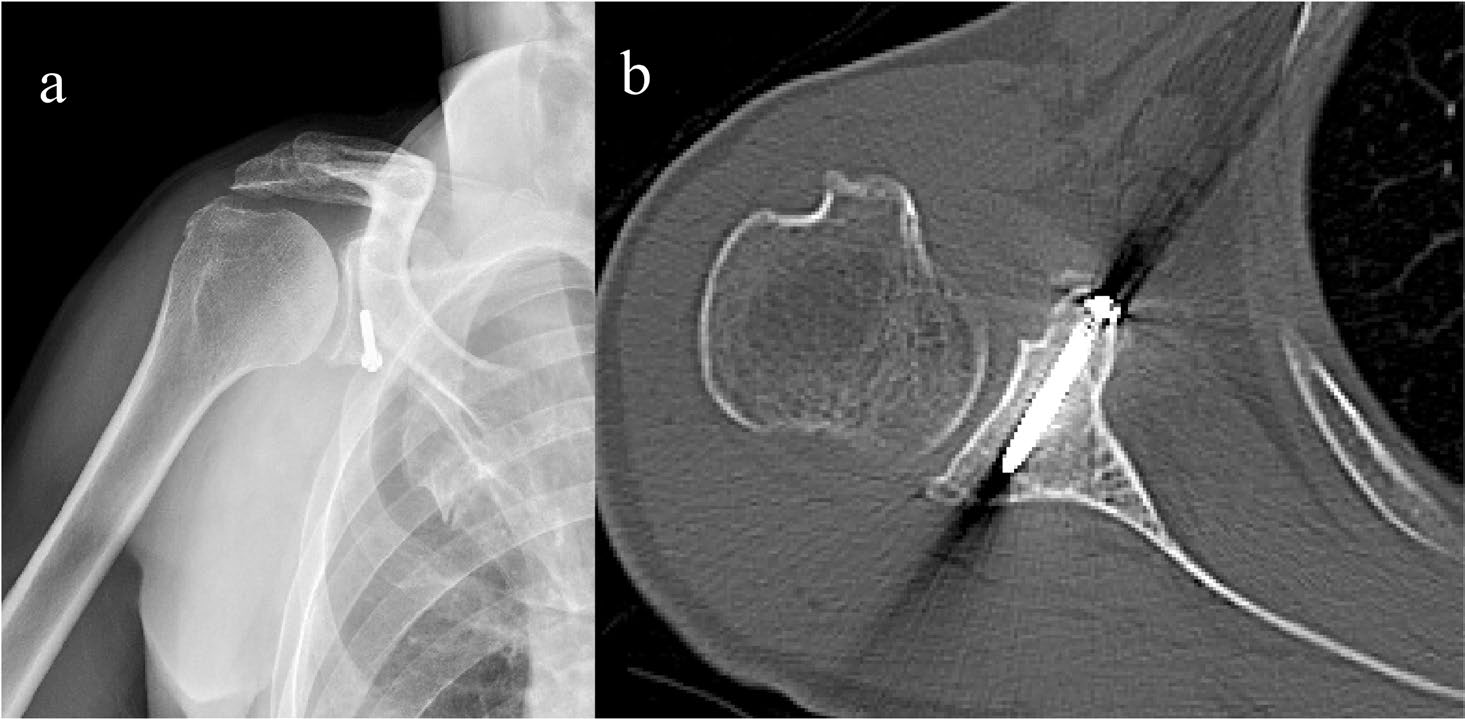
Preoperative imaging findings. (a) Plain radiograph showing irregularity of the greater tuberosity and mild acromial acetabularization without osteonecrotic change. (b) CT demonstrating bony union of prior coracoid transfer with embedded screw fixation.

**FIGURE 2:**
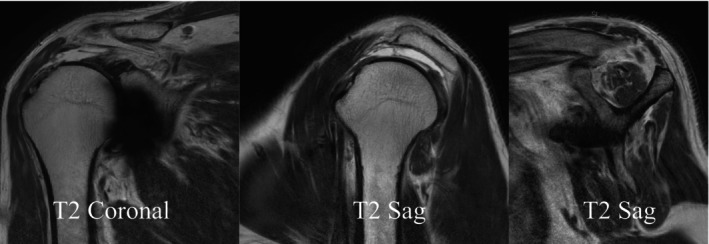
Preoperative MRI showing a large to massive rotator cuff tear involving the supraspinatus and infraspinatus with mild muscle atrophy, indicating an irreparable cuff tear.

Plain radiographs showed irregularity of the greater tuberosity and mild acromial acetabularization without evidence of osteonecrosis (Figure 1a). Computed tomography demonstrated bony union of the prior coracoid transfer with retained screw fixation (Figure 1b). MRI revealed a large to massive tear of the supraspinatus and infraspinatus with only mild muscle atrophy (Figure 2), but technical difficulty of repair was anticipated due to retained hardware and altered anatomy, leading to the decision to perform SCR.

## 3 Differential Diagnosis, Investigations, and Treatment

**FIGURE 3:**
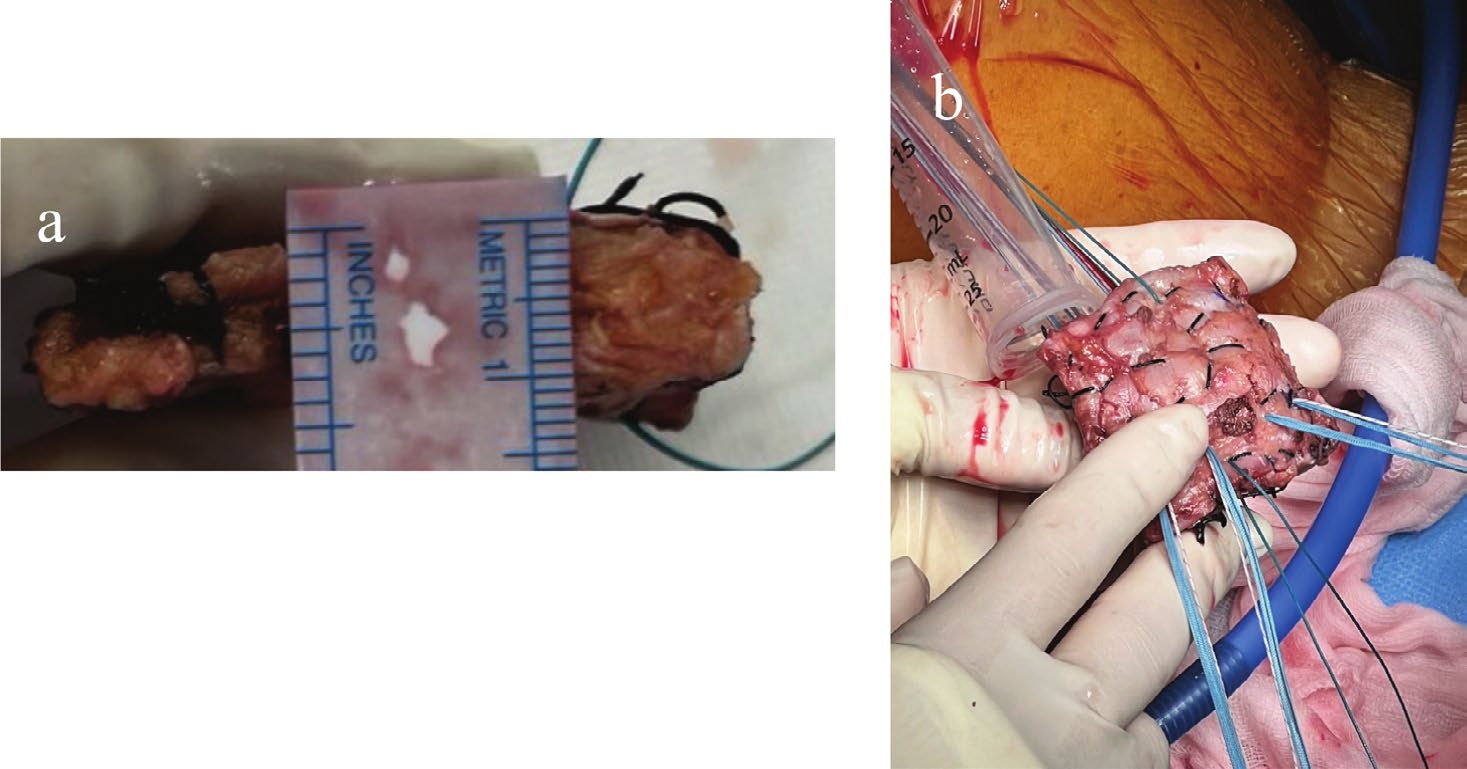
Arthroscopic superior capsule reconstruction. (a) Intraoperative view of fascia lata autograft (50 × 35 × 10 mm) used for SCR. (b) Schematic illustration of anchor placement (glenoid and humeral sides).

The rotator cuff tear involved a large to massive tear of the supraspinatus and infraspinatus tendons and was considered only partially reparable. Arthroscopic SCR was performed using a fascia lata autograft (50 × 35 × 10 mm) (Figure 3a). Two 4.5‐mm PEEK anchors were inserted on the glenoid side, and four anchors were inserted on the humeral side (Figure 3b). The long head of the biceps tendon was treated with tenotomy, and the remaining rotator cuff tissue was sutured side‐to‐side to the graft. Irrigation pressure was maintained at 50–70 mmHg, and no metallic anchors or epinephrine‐containing irrigation fluid were used. The total operative time was 2 h and 56 min, with minimal blood loss.

**FIGURE 4:**
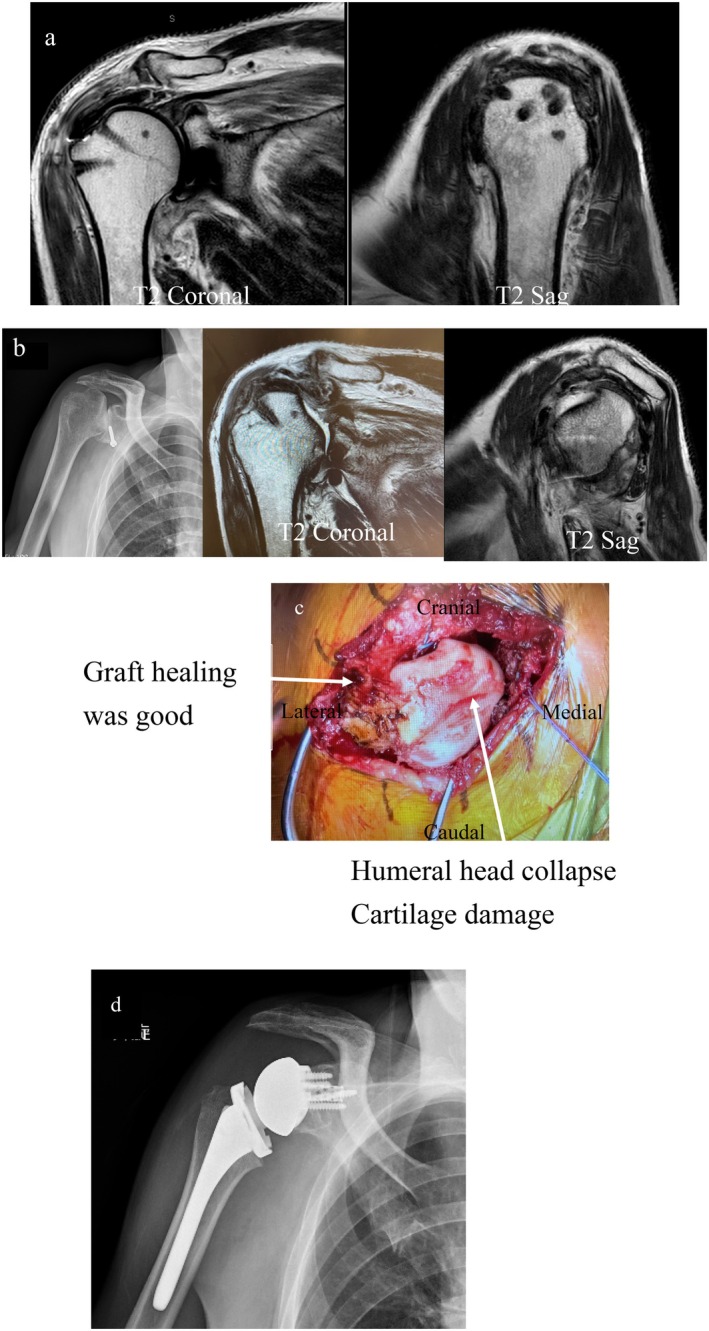
Postoperative course and RTSA. (a) MRI at 3 months confirming intact graft. (b) Radiograph at 7 months showing humeral head collapse (Cruess stage IV). (c) Intraoperative photo demonstrating humeral head collapse with preserved graft incorporation. (d) Postoperative radiograph after reverse total shoulder arthroplasty (RTSA).

The arm was immobilized in an abduction brace for 4 weeks, followed by passive motion from Week 2, active‐assisted motion at Week 4, and active motion at Week 6. At 3 months postoperatively, MRI confirmed intact graft healing (Figure 4a). At 4 months, the patient reported pain relief; however, objective improvement in shoulder range of motion was limited, and subtle stiffness persisted. At 6 months, shoulder pain recurred abruptly with progressive crepitus and decreased range of motion.

Given the abrupt recurrence of pain, the differential diagnosis included graft failure, postoperative infection, and osteonecrosis of the humeral head. Radiographs and MRI at 7 months demonstrated humeral head collapse consistent with Cruess stage IV osteonecrosis, while the SCR graft remained intact (Figure 4b). Laboratory findings, including inflammatory markers, showed no evidence of infection, and intraoperative findings later confirmed noninfectious osteonecrosis.

Because conservative treatment failed, RTSA was performed 10 months after SCR. A deltopectoral approach was used. Intraoperatively, the humeral head showed subchondral collapse and cartilage loss, while the fascia lata graft was well incorporated and intact (Figure 4c). The anterior circumflex humeral vessels were not identifiable. Histopathological examination confirmed osteonecrosis with degenerative osteoarthritis. Details of implant positioning and operative technique are omitted, as they are not central to the purpose of this report (Figure 4d).

## 4 Conclusion and Results

At 1 year post‐RTSA, the patient had marked pain relief and improved function, with forward elevation 120°, abduction 120°, external rotation 30°, and internal rotation to L1. The JOA score improved from 47 to 70 points and the VAS score from 9 to 1.5. He returned to his work as a carpenter.

## 5 Discussion

This case represents an exceptionally rare postoperative complication following arthroscopic superior capsule reconstruction (SCR). Although rapidly destructive osteonecrosis (RDON) after SCR has rarely been described in the existing English‐language literature, definitive statements regarding its novelty should be made with caution.

In contrast, RDON of the humeral head has been reported after arthroscopic rotator cuff repair (ARCR). Dilisio et al. [1] reported an incidence of 0.28% following ARCR and proposed that disruption of the humeral head vascular supply, increased intraosseous pressure, and mechanical stress related to anchor insertion may contribute to the development of osteonecrosis. Several subsequent case reports and series have similarly documented rapidly destructive humeral head collapse after ARCR, often necessitating conversion to shoulder arthroplasty [1].

The clinical course observed in the present case—characterized by initial postoperative symptom improvement followed by rapid humeral head collapse within 6–8 months—closely mirrors the temporal pattern reported in ARCR‐related RDON cases [2, 3]. This similarity suggests that a shared pathophysiologic mechanism may underlie osteonecrosis following both ARCR and SCR, despite differences in surgical indication and technique.

The vascular supply of the humeral head is primarily derived from the anterior and posterior humeral circumflex arteries, with the arcuate artery playing a particularly important role in perfusing the anterosuperior region of the humeral head [4, 5]. Previous anatomical and clinical studies have emphasized the vulnerability of this vascular network to iatrogenic injury during shoulder surgery, especially when multiple anchors are placed in close proximity to the articular surface [6, 7].

In SCR, as in ARCR, multiple anchors are inserted into the humeral head, often near the anterosuperior region. This raises concern for cumulative microvascular compromise, particularly when combined with intraoperative factors such as sustained traction, increased intraosseous pressure, and continuous irrigation flow [2, 3]. Although irrigation pressure in the present case was maintained within a relatively safe range (50–70 mmHg), and neither metallic anchors nor epinephrine‐containing irrigation fluid were used, microcirculatory disturbance may still have occurred.

Moreover, patient‐related factors likely played a contributory role. Diabetes mellitus is a well‐recognized risk factor for microvascular disease [8], and prior coracoid transfer surgery may have further altered regional vascular anatomy. These predisposing conditions may have rendered the humeral head more susceptible to ischemic injury when exposed to additional surgical stress [7].

Once RDON develops, conservative management has consistently been shown to be ineffective. Previous reports of ARCR‐related RDON have demonstrated that rapid progression to joint collapse frequently necessitates shoulder arthroplasty for pain relief and functional restoration [1, 2]. In the present case, RTSA resulted in substantial improvement in pain and shoulder function, consistent with outcomes reported in prior literature.

From a clinical standpoint, persistent or recurrent shoulder pain, crepitus, or rapid functional deterioration occurring several months after SCR should prompt consideration of osteonecrosis, even when graft integrity appears preserved. While early‐stage diagnosis may be challenging, timely radiographic and MRI evaluation once symptoms recur or worsen is essential for accurate diagnosis and appropriate intervention.

This case highlights the importance of recognizing RDON as a potential—albeit extremely rare—postoperative complication following SCR. Further accumulation of similar cases and comparative studies with ARCR‐related RDON are necessary to clarify risk factors, preventive strategies, and optimal management.

## Author Contributions

**Suguru Mikami:** conceptualization, resources. **Yohei Shimada:** methodology, project administration, writing – original draft, writing – review and editing. **Yosuke Kano:** data curation, software. **Shu Somemura:** formal analysis, supervision. **Koh Terauchi:** funding acquisition, validation. **Naoki Haraguchi:** investigation, visualization.

## Funding

The authors have nothing to report.

## Ethics Statement

This report was approved by the authors' affiliated institution.

## Consent

Written informed consent was obtained from the patient for publication of this case report and accompanying images.

## Conflicts of Interest

The authors declare no conflicts of interest.

## Data Availability

The data that support the findings of this study are available from the corresponding author upon reasonable request.
